# Aromatase Gene Polymorphisms Are Associated with Survival among Patients with Cardiovascular Disease in a Sex-Specific Manner

**DOI:** 10.1371/journal.pone.0015180

**Published:** 2010-12-10

**Authors:** Amber L. Beitelshees, Julie A. Johnson, Megan L. Hames, Yan Gong, Rhonda M. Cooper-DeHoff, Jun Wu, Sharon Cresci, Cynthia X. Ma, Carl J. Pepine, Michael A. Province, John A. Spertus, Howard L. McLeod

**Affiliations:** 1 Division of Endocrinology, Diabetes and Nutrition, University of Maryland School of Medicine, College Park, Maryland, United States of America; 2 Department of Pharmacotherapy and Translational Research and Center for Pharmacogenomics, University of Florida College of Pharmacy, Gainesville, Florida, United States of America; 3 Division of Statistical Genomics, Washington University School of Medicine, St. Louis, Missouri, United States of America; 4 Cardiovascular Division and Center for Cardiovascular Research, Washington University School of Medicine, St. Louis, Missouri, United States of America; 5 Oncology Division, Washington University School of Medicine, St. Louis, Missouri, United States of America; 6 Division of Cardiovascular Medicine, University of Florida College of Medicine, Gainesville, Florida, United States of America; 7 Mid America Heart Institute and University of Missouri Kansas City, Kansas City, Missouri, United States of America; Baylor College of Medicine, United States of America

## Abstract

**Introduction:**

*CYP19A1* encodes aromatase, the enzyme responsible for the conversion of androgens to estrogens, and may play a role in variation in outcomes among men and women with cardiovascular disease. We sought to examine genetic variation in *CYP19A1* for its potential role in sex differences in cardiovascular disease outcomes.

**Methods:**

Caucasian individuals from two independent populations were assessed: 1) a prospective cohort of patients with acute coronary syndromes with 3-year mortality follow-up (n = 568) and 2) a nested case-control study from a randomized, controlled trial of hypertension patients with stable coronary disease in which the primary outcome was death, nonfatal myocardial infarction (MI) or nonfatal stroke (n = 619). Six *CYP19A1* SNPs were genotyped (-81371 C>T, -45965 G>C, M201T, R264C, 80 A>G, and +32226 G>A). The sex*genotype interaction term was assessed for the primary outcome and compared by genotype in men and women when a significant interaction term was identified.

**Results:**

We identified a significant interaction between -81371 C>T and sex (p = 0.025) in the ACS population. The variant allele was associated with a 78% increase in mortality in men (HR 1.78, 95% confidence interval [CI] 1.08-2.94) and a nonsignificant 42% decrease in mortality among women (HR 0.58, 95% CI 0.22-1.54). We identified a similar association in the hypertensive CAD group, the -81371 C>T*sex interaction term was p<0.0001, with an associated 65% increase in death, MI, or stroke (HR 1.65, 95% CI 1.00-2.73) in men and a 69% decrease (HR 0.31, 95% CI 0.16-0.6) in women.

**Conclusions:**

Using two independent populations, this study is the first to document a significant interaction between *CYP19A1* genotype and sex on cardiovascular outcomes. These findings could illuminate potential mechanisms of sex differences in cardiovascular disease outcomes**.**

## Introduction

Aromatase is the enzyme that catalyzes the conversion of androgens to estrogens and is the primary pathway of estrogen production in men and post-menopausal women. It is encoded for by the *CYP19A1* gene located on chromosome 15q21.1. Many investigators have evaluated the role of *CYP19A1* polymorphisms in breast cancer, endometrial cancer, prostate cancer, bone health, Alzheimer's disease, obesity, and hormonal status. Recently, *CYP19A1* has also been investigated for its role in blood pressure regulation.[1], [2] Two studies have noted an association between *CYP19A1* single nucleotide polymorphisms (SNPs) and blood pressure in women, but not in men.[1], [2] However, whether differences in blood pressure by *CYP19A1* genotype translate into sex-specific differences in outcomes has not been assessed. This question is quite relevant given the differences in cardiometabolic risk factors between men and women and the fact that some of these differences may be related to sex hormones.[3], [4] Furthermore, uncertainty about the role of reproductive hormones in cardiovascular health (as evidenced by divergent results from epidemiological and clinical trials of hormone replacement therapy) necessitates careful consideration of sexual dimorphism when assessing the impact of hormone-related gene variants in cardiovascular risk.

Given the postulated role of estrogens and androgens on cardiovascular health and sex-related differences in cardiovascular disease risk factors, we hypothesized that *CYP19A1* polymorphisms would be associated with outcomes in cardiovascular disease. We sought to examine both the overall effect of *CYP19A1* and to explicitly test for gene*sex interactions and sex-specific differences in these genetic variants and outcome using two independent coronary artery disease (CAD) populations, one with acute coronary syndromes (ACS) and one with stable CAD.

## Methods

### Patient Populations

The methods of the first population (The INvestigation oF Outcomes from acute coronary syndRoMes study [INFORM]), a prospective cohort of patients with acute coronary syndromes (ACS) have been previously described.[5] Briefly, patients admitted to one of two Kansas City hospitals with a confirmed ACS were enrolled. Myocardial infarction (MI) was defined by elevated troponin level in combination with chest pain symptoms or electrocardiographic findings (ST-segment elevation or non-ST-segment elevation) consistent with MI. Unstable angina was defined by a negative troponin level and any one of the following: new-onset angina (<2 months), prolonged angina (>20 minutes) at rest, recent worsening angina, or angina that occurred within 2 weeks of MI.[6] Participating patients were interviewed during their admission in order to obtain demographic characteristics. Chart abstractions were performed in order to obtain medical histories, detailed medication histories, laboratory histories, and information regarding inpatient care at time of initial event. All-cause mortality assessment was made using the Social Security Administration Death Master File (http://www.ntis.gov/products/ssa-dmf.asp).

The second population assessed consisted of the case-control group of the INternational VErapamil SR/trandolapril Study genetic substudy (INVEST-GENES), which has also been previously described.[7], [8] Patients with hypertension and stable coronary artery disease were randomized to either a beta-blocker-based or calcium antagonist-based treatment regimen with additional anti-hypertensive medications added, as necessary, to control blood pressure. The primary outcome of the study was time to first occurrence of death, nonfatal myocardial infarction (MI), or nonfatal stroke. The nested-case control group was created by using the 258 subjects from the genetic subset who experienced a primary outcome event as cases and age-, sex-, and race-frequency-matching 781 individuals who did not experience the primary outcome as controls.

Both INFORM and INVEST were multi-ethnic but only Caucasian individuals were used for these analyses (568/723 for INFORM and 619/1054 for INVEST).

INFORM was approved by the University of Missouri- Kansas City Adult Health Sciences Institutional Review Board and INVEST was approved by the University of Florida Institutional Review Board-01. Written informed consent was obtained from all participants.

### Genotyping

**Figure 1:**
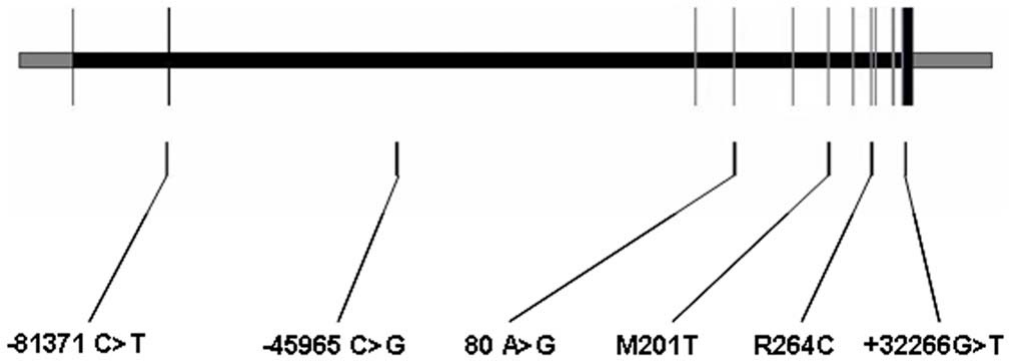
*CYP19A1* schematic with SNP locations. Exons are shown as vertical lines (noncoding in black coding in grey), introns are shown as the horizontal space between exons. SNP positions are indicated by vertical lines below the figure.

Genomic DNA was isolated from whole blood samples (for INFORM) or buccal cells (for INVEST) using standard methods. Six single nucleotide polymorphisms (SNPs) with minor allele frequencies greater than 0.04 were chosen based on prior resequencing data and functional studies (for nonsynonymous SNPs) and to emphasize SNPs with putative functional significance (promoter, synonymous, and 3′ untranslated region SNPs).[9] The SNPs consisted of two located in the promoter region: -81371 C>T (rs4774585) and -45965 G>C (rs936308); two nonsynonymous SNPs: M201T (rs28757184) and R264C (rs700519); one synonymous SNP: 80 A>G (rs700518); and one SNP located in the 3′ UTR: +32226 G>T (rs4646) (Figure 1). Genotyping was conducted using pyrosequencing (PSQ HS 96) or Taqman (ABI 7700). The PCR primers and conditions are available upon request. PCR reactions for pyrosequencing were carried out using 10 µL ABI taq master mix, 0.5 µL (1 µmol) forward and reverse primer, 10 ng DNA, and 8 µL water. Taqman assays (C__27892984_10, C___1664163_10, C___8794675_30, C___8234730_1, and C__25972590_30) were carried out according to manufacturers' instructions.

### Statistical Analysis

Baseline characteristics were compared by ANOVA, student's t-test or chi-square tests as appropriate. Allele frequencies were calculated by allele counting and deviations from Hardy-Weinberg equilibrium were assessed using chi-square tests. For the INFORM cohort, Cox proportional hazards models were developed that included the following pre-specified covariates: age, sex, ACS type (ST elevation MI, non ST elevation MI, or unstable angina), coronary revascularization strategy (medical management, percutaneous coronary intervention, or coronary artery bypass graft), ejection fraction, history of heart failure, hypertension, diabetes, and *CYP19A1* genotype (according to an additive model of inheritance). Given the role of aromatase in sex hormone metabolism, genotype-by-sex interaction terms were assessed and analyses were also conducted stratified by sex if the interaction term was significant (primary analysis) and in the overall population (secondary analysis). For the INVEST case-control population, logistic regression models were developed including age, sex, BMI, blood pressure, history of MI, heart failure, diabetes, or stroke, smoking, ACE inhibitor use, diuretic use, and treatment randomization group and genotype (according to an additive model of inheritance)-by-sex interaction terms. Haplotypes were assigned using PHASE (version 2.1).

## Results

**Figure 2:**
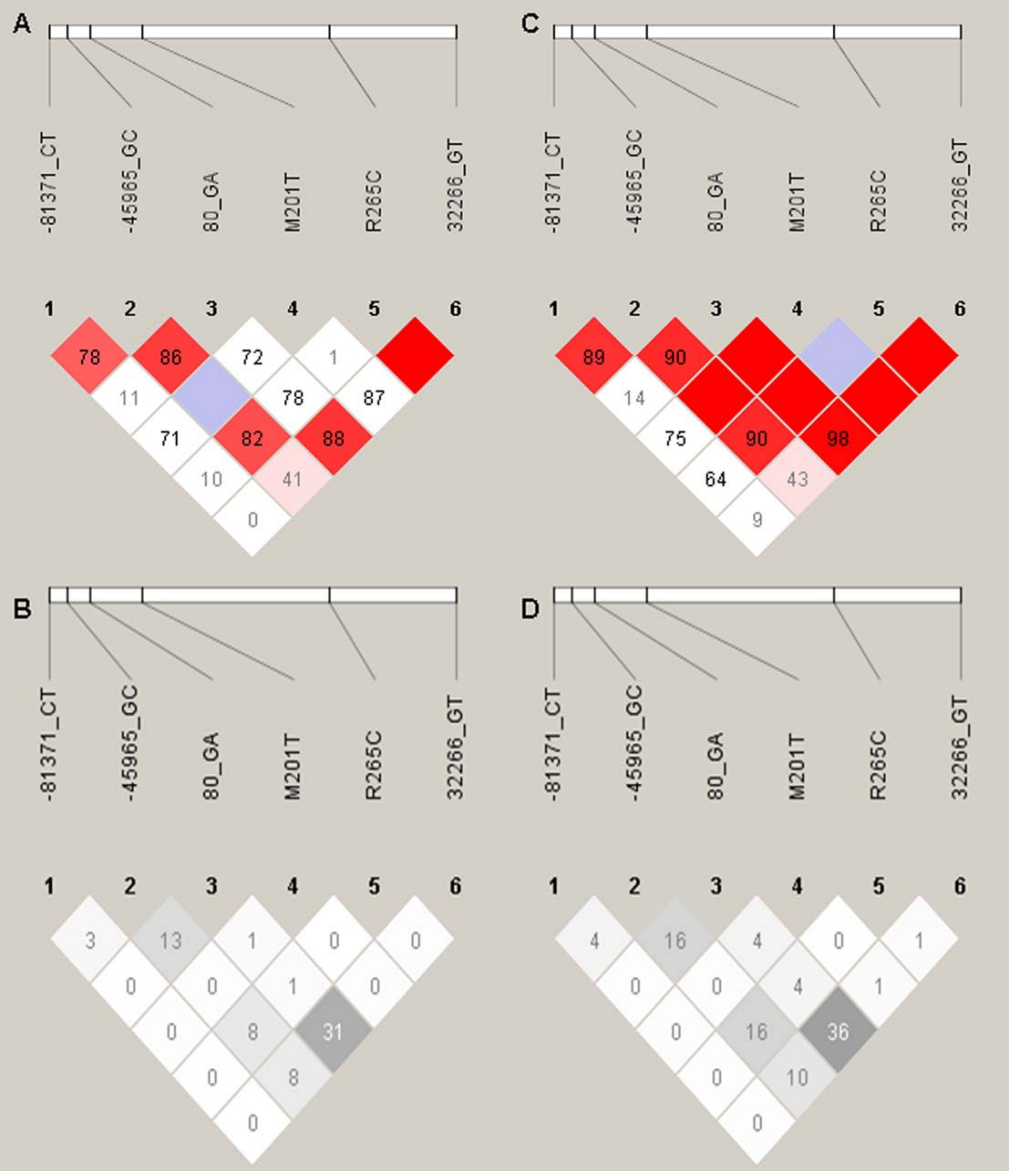
Linkage disequilibrium plots for Caucasians. Panels A and B are INFORM and panels C and D are INVEST. The colored plots show D' and the black and white plots show r^2^. Blank red boxes indicate D' of 1.0.

The first population (INFORM) consisted of 568 Caucasian ACS patients who consented to participate in the genetic analyses of this study. Genotyping was complete for 98%, 91%, 96%, 98%, 99%, and 97% for -81371 C>T, -45965 G>C, 80 A>G, M201T, R264C, and 32266 G>T, respectively. The second population (INVEST-GENES) consisted of 619 Caucasian hypertensive patients with stable coronary disease. Genotyping was complete for 87%, 96%, 97%, 98%, 96%, and 99%) for -81371 C>T, -45965 G>C, 80 A>G, M201T, R264C, and 32266 G>T, respectively. Allele frequencies for Caucasian INFORM and INVEST-GENES are shown in Table 1. As expected, no genotype frequencies differed by sex and no SNP deviated from Hardy-Weinberg equilibrium expectations (sex-stratified or overall). The linkage disequilibrium plots for INFORM and INVEST are shown in Figure 2.

**Table 1:** Allele Frequencies in INFORM and INVEST.

SNP*	INFORM	INVEST
-81371 C>T	0.23	0.22
-45965 G>C	0.15	0.17
80 G>A	0.51	0.50
M201T	0.03	0.04
R264C	0.03	0.04
32266 G>T	0.27	0.27

*SNP- single nucleotide polymorphism, presented as A>B, where B indicates minor allele.

INFORM patients were, on average, 61±12 years of age, of whom 36% were women, and 29% presented with ST elevation MI, 30% with non-ST elevation MI, and 41% with unstable angina (Table 2). Consistent with previous investigations of ACS, women were older, had higher BMI, were more likely to present with unstable angina or non-ST elevation MI and, had a greater prevalence of hypertension, heart failure, and diabetes than men (Table 2).[10], [11] The INVEST-GENES case-control patients were, on average 71±10 years of age, made up of 51% women, and 33% had a history of MI, 62% had chronic stable angina, and 49% had an abnormal angiogram that qualified them for the study (Table 2). The baseline characteristics compared by genotype and sex are shown in File S1.

**Table 2:** Baseline Characteristics- INFORM and INVEST-GENES.

	INFORM(n = 568)	INFORM Men(n = 370)	INFORM Women(n = 198)	INVEST(n = 619)	INVEST Men(n = 314)	INVEST Women(n = 305)
Age, mean ± SD, years	62±12	61±12	64±13	71±10	69±9	73±10
BMI, mean ± SD, kg/m2	29.4±5.9	29.0±5.5	30.1±6.5	28.3±5.1	28.5±4.3	28.1±5.9
*Smoking status, number (%)*						
Current	185 (33)	127 (34)	58 (29)	67 (11)	42 (13)	25 (8)
Past	220 (39)	154 (42)	66 (33)	258 (42)	162 (52)	96 (31)
Never	162 (29)	88 (24)	74 (37)	294 (48)	110 (35)	184 (60)
SBP, mean ± SD, mmHg	134±25	136±25	132±24	149±19	146±18	151±20
DBP, mean ± SD, mmHg	72±15	74±15	69±14	82±11	82±11	82±11
*ACS type, number (%)*						
ST elevation MI	174 (31)	128 (35)	46 (23)	n/a	n/a	n/a
Non-ST elevation MI	182 (32)	112 (30)	70 (35)	n/a	n/a	n/a
Unstable angina	210 (37)	130 (40)	80 (43)	n/a	n/a	n/a
Old LBB/Unknown	2 (0.4)	0 (0)	2 (1)	n/a	n/a	n/a
Chronic stable angina, number (%)	n/a	n/a	n/a	291 (47)	125 (40)	166 (54)
Unstable angina >1 mo ago, number (%)	n/a	n/a	n/a	104 (17)	74 (24)	30 (10)
Abnormal angiogram, number (%)	n/a	n/a	n/a	416 (67)	252 (80)	165 (54)
Abnormal stress test, number (%)	n/a	n/a	n/a	127 (21)	84 (27)	43 (14)
*ACS treatment strategy, number (%)*						
Medical management	162 (29)	99 (27)	63 (32)	n/a	n/a	n/a
PCI	379 (67)	252 (68)	127 (64)	n/a	n/a	n/a
CABG	27 (5)	19 (5)	8 (4)	n/a	n/a	n/a
Myocardial infarction, number (%)	183(32)	115 (31)	68 (34)	265 (43)	142 (45)	123 (40)
Hypertension, number (%)	356 (63)	208 (56)	148 (75)	619 (100)	314 (100)	305 (100)
Heart failure, number (%)	31 (5)	14 (54)	17 (129)	33 (5)	13 (4)	20 (7)
Diabetes, number (%)	137 (24)	80 (22)	57 (29)	12 (2)	5 (2)	7 (2)
Hormone replacement therapy, number (%)	55 (10)	2 (0.45)	53 (27)	81 (13)	0 (0)	81 (27)

n/a not applicable.

Adjusted hazard ratios for individual SNPs in the INFORM overall population, men, and women are shown in Table 3. We identified a significant interaction between -81371 C>T and sex in whites (p = 0.02, Table 3). In white men, the variant allele was associated with a significant increase in mortality and in women the variant allele was associated with a nonsignificant decreased risk of mortality (Table 3). In the overall INFORM population (men and women combined), -81371 was associated with a 72% increase in mortality (HR 1.72, 95% CI 1.06-2.79) and M201T was associated with a nearly 3-fold increase in mortality (HR 2.99, 95% CI 1.15-7.78). The Kaplan Meier event plots are shown in File S1. Whereas the association with -81371 C>T was divergent by sex, the association with M201T was similar in men and women (Table 3). We did not identify any significant associations for -45965 G>C, 80 G>A, or +3266 G>T with long-term mortality.

**Table 3:** Adjusted Hazard Ratios/Odds Ratios and 95% Confidence Intervals by Individual SNPs in Whites.

SNP	All whites	Genotype*sex interaction whites	White men	White women
**INFORM**				
-81371 C>T	1.72 (1.06-2.79)p = 0.03*	0.02*	1.78 (1.08-2.94)p = 0.025*	0.58 (0.22-1.54)p = 0.28
-45965 G>C	0.95 (0.55-1.63)p = 0.84	0.56		
80 G>A	1.16 (0.79-1.70)p = 0.46	n/a		
M201T	2.99 (1.15-7.78)p = 0.025*	0.91		
R264C	0.31 (0.04-2.33)p = 0.26	0.99		
32226 G>T	0.97 (0.63-1.48)p = 0.87	0.50		
**INVEST-GENES**				
-81371 C>T	0.88 (0.60-1.27)p = 0.48	7.9×10^−5^ *	1.65 (1.00-2.73)p = 0.05	0.31 (0.16-0.60)p = 0.0006*
-45965 G>C	1.10 (0.74-1.64)p = 0.62	0.018*	0.66 (0.36-1.20)p = 0.17	1.83 (1.00-3.32)p = 0.048*
80 G>A	0.99 (0.73-1.34)p = 0.94	0.33		
M201T	1.15 (0.57-2.34)p = 0.70	0.98		
R264C	1.79 (0.83-3.90)p = 0.14	0.44		
32226 G>T	1.00 (0.72-1.40)p = 0.99	0.62		

*indicates p<0.05.

n/a indicates not able to be calculated due to small numbers.

In an independent cohort with hypertension and stable CAD, we observed the same significant interaction between -81371 C>T and sex (p<0.0001, Table 3). In white men, the variant allele was associated with a 65% increase in the primary outcome (OR 1.65, 95% CI 1.00-2.73) and in women, the variant allele was associated with a 69% reduced risk of the primary outcome (OR 0.31, 95% CI 0.16-0.60) (Table 3). Unlike the ACS cohort, -81371 C>T was not significantly associated with the primary outcome in the overall population, potentially due to the higher percentage of women in INVEST-GENES than INFORM. The association with M201T and outcomes were not replicated in INVEST-GENES. The case-control status by genotype is shown in File S1.

Haplotype analysis was not consistently more informative than the individual SNP analyses (data not shown).

## Discussion

We identified a significant interaction between *CYP19A1* -81371 C>T genotype and sex on mortality after an ACS. This finding was replicated in an independent population of patients with stable CAD and hypertension. The variant allele was associated with an increase in outcomes among men and a decreased risk of outcomes in women. To our knowledge, this is the first report of such an association.

These findings markedly extend previous investigations of *CYP19A1* and cardiovascular disease. While the mechanisms of these sex-divergent genotype effects on mortality are currently unknown, previous work lends support for this phenomenon. For example, using data from the Framingham Heart Study, Peter et al found a single SNP in *CYP19A1* in which the homozygous variant genotype was associated with *higher* diastolic blood pressure in women and *lower* pulse pressure in men.[1] Additionally, Ramirez-Lorca et al reported an association with *CYP19A1* and blood pressure in women, but not men.[2] Therefore, our findings are the first of our knowledge in large cardiovascular disease populations to extend the previous sex-dependent effects of *CYP19A1* on blood pressure to adverse outcomes.

Unfortunately, we do not have access to hormone levels in these populations. However, Peter et al have previously found SNPs in CYP19A1 (including rs700518 investigated in this study) to be associated with estradiol, testosterone, and the estradiol to testosterone ratio in men, but not in women.[12] The minor allele rs700518 (A) carriers had higher estradiol, lower testosterone levels, and higher estradiol to testosterone ratios than the major (G) allele homozygotes. Therefore, taken together with previous epidemiological data, our findings in which -81371 C>T male variant carriers had increased mortality might suggest that -81371 T (or a linked SNP) is associated with lower testosterone levels and lower testosterone to estradiol ratio.{Laughlin, 2008 #19}{Peter, 2008 #18}

Our findings support several potential hypotheses as to the underlying mechanisms responsible for the observed sex differences in *CYP19A1* associations. First, sex-biased gene expression is increasingly being identified across many organisms' genomes.[13] In fact, up to 57% of genes in *Drosophila melanogaster* have been found to have sex-biased expression, many of which are expressed in reproductive tissues.[14], [15] If *CYP19A1* is susceptible to differences in expression between sexes, this could allow for the same variant to have different effects because of differences in *trans*-regulation of gene expression. A second explanation for the sex-differences we observed is different sex-dependent relationships between androgens and cardiovascular disease or insulin resistance. Hyperandrogenic conditions in women such as polycystic ovary disease have been associated with insulin resistance and an increased risk of cardiovascular death or MI.[16], [17]. In contrast, hypoandrogenism in men has been associated with insulin resistance and increased mortality.[17], [18] Therefore, our findings of a polymorphism in *CYP19A1* being associated with excess risk in men, while being protective in women, may be the result of differences in effects of sex hormones in men and women. Finally, our sex-divergent findings may result from gene-by-environment interactions. Men and women differed in their age at presentation, body mass indices, the frequency of comorbid conditions including hypertension, diabetes, and heart failure, and in the type of ACS with which they presented, all factors which were adjusted for in our analyses with the sex-by-genotype interaction still persisting. Therefore, aromatase polymorphisms may interact with any of these variables in their influence on prognosis (although we did not detect any such interactions), or they may interact with other unmeasured differences between men and women (e.g. the hormonal environment) which could result in different effects of the same genotype in men as compared with women.

### Limitations

The vast majority of the women in these studies were likely post-menopausal given that the minimum age for enrollment in INVEST was 50 (mean 72±9) and the mean age in INFORM was 62±13 years. Using an age cut-off of 55 years and hormone replacement therapy use, only 127 women in INFORM and 130 women in INVEST were less than 55 or on hormone replacement therapy. Therefore, a limitation of our study is that these small numbers prevented us from being able to conduct any meaningful analyses using hormonal status instead of just sex. Further, our findings are likely not translatable to younger, premenopausal women.

Another limitation of our study is that we do not have access to hormone levels in these populations which would be very valuable for assessing genotype associations. Last, the CYP19A1 gene is very large and we only looked at common SNPs with putative function located across the gene. We did not fully tag the gene and hence, the genomic coverage is actually quite low. Therefore, we cannot rule out other associations with unmeasured SNPs nor can we determine the causative functional SNPs based on the observed associations. Last, there is the potential for type I error.

### Conclusions

We have identified a striking association between *CYP19A1* genotype and outcomes in a group of post-ACS patients which was divergent in men and women. We replicated these findings in an independent population of hypertensive patients with stable coronary disease. In men, the *CYP19A1* -81371 C>T variant was associated with an increased risk of adverse outcomes, whereas in women, the variant was associated with a reduced risk for adverse outcomes. These findings could have broad implications for understanding sex differences in cardiovascular disease, the treatment of CVD, the therapeutic uses of aromatase inhibitors, and risk assessment for CVD prognosis.

## Supporting Information

File S1 Includes tables of baseline characteristics by genotype and sex for INFORM and INVEST, table of case/control status by sex and genotype in INVEST, and Kaplan Meier plots of cumulative mortality incidence by -81371 C>T genotype and sex.
(DOC)
