# Supplementary material for: Aromatase Gene Polymorphisms Are Associated with Survival among Patients with Cardiovascular Disease in a Sex-Specific Manner
Source: PLoS One. 2010 Dec 10;5(12):e15180. doi: 10.1371/journal.pone.0015180 (PMC3000815; doi:10.1371/journal.pone.0015180)

**Supporting Information S1**

Supplemental Table 1. INFORM baseline characteristics by -81371 C>T and sex

|  | **-81371 C/C male (n=217)** | **-81371 C/T male (n=125)** | **-81371 T/T male (n=21)** | **-81371 C/C female (n=117)** | **-81371 C/T female (n=65)** | **-81371 T/T female (n=9)** |
| --- | --- | --- | --- | --- | --- | --- |
| Age, mean ± SD, years | 63 ± 12 | 63 ± 12 | 66 ± 11 | 56 ± 12 | 57 ± 14 | 58 ± 9 |
| BMI, mean ± SD, kg/m2 | 28.9 ± 5.6 | 29.1 ± 5.7 | 28.2 ± 6.0 | 31.4 ± 5.9 | 30.7 ± 8.1 | 33.2 ± 3.7 |
| *Smoking status, number (%)*  Current | 72 (33) | 48 (39) | 4 (19) | 37 (32) | 17 (26) | 1 (11) |
| Past | 96 (44) | 46 (37) | 8 (38) | 38 (32) | 21 (32) | 5 (56) |
| Never | 49 (23) | 30 (24) | 9 (43) | 42 (36) | 27 (42) | 3 (33) |
| SBP, mean ± SD, mmHg | 135 ± 24 | 137 ± 25 | 142 ± 27 | 126 ± 22 | 131 ± 28 | 143 ± 24 |
| DBP, mean ± SD, mmHg | 72 ± 15 | 76 ± 15 | 71 ± 15 | 67 ± 16 | 68 ± 12 | 78 ± 17 |
| *ACS type, number (%)*  STEMI | 72 (33) | 46 (37) | 7 (33) | 29 (25) | 14 (22) | 2 (22) |
| NSTEMI | 73 (34) | 34 (27) | 5 (24) | 41 (35) | 24 (37) | 2 (22) |
| UA | 72 (33) | 45 (36) | 9 (43) | 46 (39) | 26 (40) | 5 (56) |
| Old LBB/Unknown | 0 (0) | 0 (0) | 0 (0) | 1 (1) | 1 (2) | 0 (0) |
| *ACS treatment strategy, number (%)*  Medical  management | 52 (24) | 34 (27) | 8 (38) | 35 (30) | 17 (26) | 4 (44) |
| PCI | 157 (72) | 83 (66) | 12 (57) | 77 (66) | 46 (71) | 4 (44) |
| CABG | 8 (4) | 8 (6) | 1 (5) | 5 (4) | 2 (3) | 1 (11) |
| Myocardial infarction, number (%) | 60 (28) | 43 (34) | 9 (43) | 35 (30) | 25 (39) | 4 (44) |
| Hypertension, number (%) | 126 (57) | 66 (53) | 16 (76) | 82 (70) | 50 (77) | 9 (100) |
| Heart failure, number (%) | 8 (4) | 5 (4) | 1 (5) | 4 (3) | 7 (11) | 4 (44) |
| Diabetes, number (%) | 48 (22) | 26 (21) | 4 (19) | 34 (29) | 14 (22) | 4 (44) |
| Hormone replacement therapy, number (%) | 2 (1) | 0 (0) | 0 (0) | 35 (30) | 13 (20) | 3 (33) |

STEMI- ST elevation myocardial infarction

NSTEMI- non-ST elevation myocardial infarction

UA- unstable angina

Supplemental Table 2. INFORM baseline characteristics by -45965 G>C and sex

|  | **-45965 G/G male (n=245)** | **-45965 G/C male (n=81)** | **-45965 C/C male (n=10)** | **-45965 G/G female (n=131)** | **-45965 G/C female (n=45)** | **-45965 C/C female (n=4)** |
| --- | --- | --- | --- | --- | --- | --- |
| Age, mean ± SD, years | 63 ± 12 | 62 ± 12 | 70 ± 13 | 56 ± 12 | 57 ± 14 | 65 ± 24 |
| BMI, mean ± SD, kg/m2 | 29.1 ± 5.6 | 28.3 ± 5.1 | 28.3 ± 5.5 | 31.5 ± 6.3 | 32.2 ± 6.6 | 28.5 ± 4.4 |
| *Smoking status, number (%)*  Current | 86 (35) | 31 (38) | 2 (20) | 39 (30) | 12 (27) | 2 (50) |
| Past | 97 (40) | 28 (35) | 6 (60) | 39 (30) | 18 (40) | 1 (25) |
| Never | 61 (25) | 22 (27) | 2 (20) | 53 (40) | 15 (33) | 1 (25) |
| SBP, mean ± SD, mmHg | 136 ± 25 | 134 ± 25 | 138 ± 27 | 129 ± 23 | 125 ± 25 | 114 ± 33 |
| DBP, mean ± SD, mmHg | 73 ± 14 | 74 ± 17 | 73 ± 15 | 68 ± 16 | 67 ± 10 | 67 ± 20 |
| *ACS type, number (%)*  STEMI | 85 (35) | 31 (38) | 4 (40) | 33 (25) | 11 (24) | 0 (0) |
| NSTEMI | 77 (31) | 23 (28) | 3 (30) | 45 (34) | 14 (31) | 4 (100) |
| UA | 83 (34) | 27 (33) | 3 (30) | 51 (39) | 20 (44) | 0 (0) |
| Old  LBB/Unknown | 0 (0) | 0 (0) | 0 (0) | 2 (2) | 0 (0) | 0 (0) |
| *ACS treatment strategy, number (%)*  Medical  management | 68 (28) | 17 (21) | 2 (20) | 46 (33) | 16 (36) | 0 (0) |
| PCI | 161 (66) | 63 (78) | 8 (80) | 83 (63) | 27 (60) | 3 (75) |
| CABG | 16 (7) | 1 (1) | 0 (0) | 5 (4) | 2 (4) | 1 (25) |
| Myocardial infarction, number (%) | 82 (33) | 19 (24) | 3 (30) | 44 (34) | 16 (36) | 1 (25) |
| Hypertension, number (%) | 133 (54) | 45 (56) | 7 (70) | 106 (81) | 28 (62) | 1 (25) |
| Heart failure, number (%) | 10 (4) | 1 (1) | 0 (0) | 8 (6) | 5 (11) | 1 (25) |
| Diabetes, number (%) | 53 (22) | 14 (17) | 5 (50) | 44 (34) | 9 (20) | 0 (0) |
| Hormone replacement therapy, number (%) | 0 | 0 | 1 (10) | 37 (28) | 10 (22) | 2 (50) |

STEMI- ST elevation myocardial infarction

NSTEMI- non-ST elevation myocardial infarction

UA- unstable angina

Supplemental Table 3. INFORM baseline characteristics by 80 G>A and sex

|  | **80 G/G male (n=88)** | **80 G/A male (n=175)** | **80 A/A male (n=88)** | **80 G/G female (n=45)** | **80 G/A female (n=91)** | **80 A/A female (n=57)** |
| --- | --- | --- | --- | --- | --- | --- |
| Age, mean ± SD, years | 63 ± 12 | 63 ± 12 | 63 ± 12 | 57 ± 14 | 57 ± 13 | 54 ± 10 |
| BMI, mean ± SD, kg/m2 | 29.6 ± 5.9 | 28.6 ± 5.6 | 28.9 ± 5.3 | 30.4 ± 7.0 | 32.0 ± 6.6 | 31.5 ± 5.9 |
| *Smoking status, number (%)*  Current | 32 (36) | 58 (33) | 32 (37) | 16 (36) | 27 (30) | 13 (23) |
| Past | 30 (34) | 81 (46) | 33 (38) | 13 (29) | 35 (39) | 16 (28) |
| Never | 26 (30) | 36 (21) | 22 (25) | 16 (36) | 29 (32) | 28 (49) |
| SBP, mean ± SD, mmHg | 136 ± 24 | 136 ± 25 | 135 ± 25 | 114 ± 18 | 133 ± 27 | 127 ± 19 |
| DBP, mean ± SD, mmHg | 73 ± 14 | 74 ± 15 | 72 ± 15 | 61 ± 11 | 71 ± 15 | 69 ± 11 |
| *ACS type, number (%)*  STEMI | 26 (30) | 70 (40) | 31 (35) | 8 (18) | 20 (22) | 17 (30) |
| NSTEMI | 31 (35) | 43 (25) | 28 (32) | 19 (42) | 30 (33) | 19 (33) |
| UA | 31 (35) | 62 (35) | 29 (33) | 18 (40) | 40 (44) | 20 (35) |
| Old  LBB/Unknown | 0 (0) | 0 (0) | 0 (0) | 0 (0) | 1 (1) | 1 (2) |
| *ACS treatment strategy, number (%)*  Medical  management | 23 (26) | 46 (26) | 23 (26) | 13 (29) | 34 (37) | 14 (25) |
| PCI | 62 (70) | 121 (69) | 58 (66) | 28 (62) | 55 (60) | 41 (72) |
| CABG | 3 (3) | 8 (5) | 7 (8) | 4 (9) | 2 (2) | 2 (4) |
| Myocardial infarction, number (%) | 26 (30) | 54 (31) | 31 (35) | 16 (36) | 31 (34) | 20 (35) |
| Hypertension, number (%) | 52 (59) | 97 (55) | 45 (51) | 30 (67) | 67 (74) | 46 (81) |
| Heart failure, number (%) | 2 (2) | 5 (3) | 6 (7) | 6 (13) | 7 (8) | 2 (4) |
| Diabetes, number (%) | 21 (24) | 37 (21) | 17 (19) | 14 (31) | 25 (27) | 17 (30) |
| Hormone replacement therapy, number (%) | 1 (1) | 0 (0) | 0 (0) | 10 (22) | 29 (32) | 13 (23) |

STEMI- ST elevation myocardial infarction

NSTEMI- non-ST elevation myocardial infarction

UA- unstable angina

Supplemental Table 4. INFORM baseline characteristics by M201T and sex

|  | **M201M male (n=346)** | **M201T male (n=19)** | **M201M female (n=179)** | **M201T female (n=15)** |
| --- | --- | --- | --- | --- |
| Age, mean ± SD, years | 63 ± 12 | 62 ± 9 | 56 ± 12 | 60 ± 15 |
| BMI, mean ± SD, kg/m2 | 29.0 ± 5.8 | 30.0 ± 4.7 | 31.6 ± 6.5 | 32.6 ± 3.9 |
| *Smoking status, number (%)*  Current | 122 (35) | 4 (21) | 51 (28) | 5 (33) |
| Past | 142 (41) | 10 (53) | 61 (34) | 3 (20) |
| Never | 81 (23) | 5 (26) | 67 (37) | 7 (47) |
| SBP, mean ± SD, mmHg | 135 ± 25 | 137 ± 26 | 126 ± 22 | 146 ± 36 |
| DBP, mean ± SD, mmHg | 73 ± 15 | 77 ± 14 | 67 ± 15 | 66 ± 19 |
| *ACS type, number (%)*  STEMI | 118 (34) | 9 (47) | 43 (24) | 3 (20) |
| NSTEMI | 105 (30) | 4 (21) | 64 (36) | 4 (27) |
| UA | 123 (36) | 6 (32) | 71 (40) | 8 (53) |
| Old LBB/Unknown | 0 (0) | 0 (0) | 1 (1) | 0 (0) |
| *ACS treatment strategy, number (%)*  Medical  management | 92 (27) | 6 (32) | 59 (33) | 3 (20) |
| PCI | 237 (69) | 11 (58) | 113 (63) | 11 (73) |
| CABG | 17 (5) | 2 (11) | 7 (4) | 1 (7) |
| Myocardial infarction, number (%) | 238 (31) | 13 (32) | 62 (35) | 4 (27) |
| Hypertension, number (%) | 195 (56) | 10 (53) | 130 (73) | 14 (93) |
| Heart failure, number (%) | 11 (3) | 2 (11) | 17 (10) | 0 (0) |
| Diabetes, number (%) | 78 (23) | 1 (5) | 52 (29) | 5 (33) |
| Hormone replacement therapy, number (%) | 2 (1) | 0 (0) | 45 (25) | 6 (40) |

*No T201T individuals

STEMI- ST elevation myocardial infarction

NSTEMI- non-ST elevation myocardial infarction

UA- unstable angina

Supplemental Table 5. INFORM baseline characteristics by R264C and sex

|  | **R264R male (n=352)** | **R264C male (n=16)** | **R264R female (n=186)** | **R264C female (n=12)** |
| --- | --- | --- | --- | --- |
| Age, mean ± SD, years | 63 ± 12 | 63 ± 14 | 56 ± 12 | 66 ± 13 |
| BMI, mean ± SD, kg/m2 | 29.1 ± 5.7 | 28.0 ± 5.4 | 31.5 ± 6.4 | 31.2 ± 5.7 |
| *Smoking status, number (%)*  Current | 117 (33) | 9 (56) | 55 (30) | 3 (25) |
| Past | 149 (42) | 4 (25) | 61 (33) | 5 (42) |
| Never | 85 (24) | 3 (19) | 70 (38) | 4 (33) |
| SBP, mean ± SD, mmHg | 135 ± 24 | 146 ± 28 | 129 ± 24 | 119 ± 19 |
| DBP, mean ± SD, mmHg | 73 ± 15 | 78 ± 14 | 67 ± 15 | 70 ± 9 |
| *ACS type, number (%)*  STEMI | 120 (34) | 7 (44) | 44 (24) | 2 (17) |
| NSTEMI | 109 (31) | 3 (19) | 66 (35) | 4 (33) |
| UA | 123 (35) | 6 (38) | 74 (40) | 6 (50) |
| Old  LBB/Unknown | 0 (0) | 0 (0) | 2 (1) | 0 (0) |
| *ACS treatment strategy, number (%)*  Medical  management | 94 (27) | 4 (25) | 61 (33) | 2 (17) |
| PCI | 240 (68) | 11 (69) | 117 (63) | 10 (83) |
| CABG | 18 (5) | 1 (6) | 8 (4) | 0 (0) |
| Myocardial infarction, number (%) | 110 (31) | 5 (31) | 65 (35) | 3 (25) |
| Hypertension, number (%) | 198 (56) | 10 (63) | 140 (75) | 8 (67) |
| Heart failure, number (%) | 14 (4) | 0 (0) | 13 (7) | 4 (33) |
| Diabetes, number (%) | 78 (22) | 2 (13) | 54 (29) | 3 (25) |
| Hormone replacement therapy, number (%) | 2 (1) | 0 (0) | 50 (27) | 3 (25) |

*No C264C individuals

STEMI- ST elevation myocardial infarction

NSTEMI- non-ST elevation myocardial infarction

UA- unstable angina

Supplemental Table 6. INFORM baseline characteristics by 32226 G>T and sex

|  | **32266 G/G male (n=192)** | **32266 G/T male (n=135)** | **32266 T/T male (n=31)** | **32266 G/G female (n=100)** | **32266 G/T female (n=80)** | **32266 T/T female (n=12)** |
| --- | --- | --- | --- | --- | --- | --- |
| Age, mean ± SD years | 64 ± 13 | 62 ± 12 | 65 ± 11 | 56 ± 12 | 58 ± 13 | 50 ± 9.8 |
| BMI, mean ± SD, kg/m2 | 28.8 ± 5.8 | 29.2 ± 5.7 | 30.0 ± 5.3 | 31.2 ± 5.5 | 32.8 ± 7.3 | 27.9 ± 6.1 |
| *Smoking status, number (%)*  Current | 66 (35) | 48 (36) | 8 (26) | 28 (28) | 23 (29) | 6 (50) |
| Past | 76 (40) | 59 (44) | 13 (42) | 33 (33) | 29 (36) | 3 (25) |
| Never | 49 (26) | 28 (21) | 10 (32) | 39 (39) | 28 (35) | 3 (25) |
| SBP, mean ± SD, mmHg | 136 ± 25 | 134 ± 24 | 138 ± 25 | 126 ± 22 | 132 ± 28 | 120 ± 21 |
| DBP, mean ± SD, mmHg | 73 ± 15 | 74 ± 15 | 72 ± 12 | 66 ± 16 | 69 ± 14 | 64 ± 13 |
| *ACS type, number (%)*  STEMI | 70 (36) | 43 (32) | 12 (39) | 25 (25) | 15 (19) | 5 (42) |
| NSTEMI | 59 (31) | 43 (32) | 8 (26) | 32 (32) | 31 (39) | 6 (50) |
| UA | 63 (33) | 49 (36) | 11 (36) | 42 (42) | 33 (41) | 1 (8) |
| Old LBB/Unknown | 0 (0) | 0 (0) | 0 (0) | 1 (1) | 1 (1) | 0 (0) |
| *ACS treatment strategy, number (%)*  Medical  management | 52 (27) | 33 (24) | 8 (26) | 31 (31) | 26 (33) | 3 (25) |
| PCI | 129 (67) | 95 (70) | 23 (74) | 64 (64) | 51 (64) | 9 (75) |
| CABG | 11 (6) | 7 (5) | 0 (0) | 5 (5) | 3 (4) | 0 (0) |
| Myocardial infarction, number (%) | 57 (30) | 40 (30) | 14 (45) | 36 (36) | 25 (31) | 3 (25) |
| Hypertension, number (%) | 101 (53) | 80 (59) | 20 (65) | 77 (77) | 59 (74) | 7 (58) |
| Heart failure, number (%) | 9 (5) | 2 (1) | 3 (10) | 5 (5) | 10 (13) | 0 (0) |
| Diabetes, number (%) | 38 (20) | 30 (22) | 10 (32) | 26 (26) | 26 (33) | 2 (17) |
| Hormone replacement therapy, number (%) | 0 (0) | 1 (1) | 1 (3) | 27 (27) | 24 (30) | 2 (17) |

STEMI- ST elevation myocardial infarction

NSTEMI- non-ST elevation myocardial infarction

UA- unstable angina

Supplemental Table 7. INVEST baseline characteristics by -81371 C>T and sex

|  | **-81371 C/C male (n=166)** | **-81371 C/T male (n=92)** | **-81371 TT male (n=13)** | **-81371 C/C female**  **(n=158)** | **-81371 C/T female (n=96)** | **-81371 T/T female (n=14)** |
| --- | --- | --- | --- | --- | --- | --- |
| Age, mean ± SD, years | 70 ± 9 | 69 ± 9 | 67 ± 11 | 73 ± 10 | 73 ± 10 | 73 ± 10 |
| BMI, mean ± SD, kg/m2 | 28.4 ± 4.3 | 28.7 ± 4.5 | 30.8 ± 5.0 | 27.8 ± 5.9 | 28.2 ± 5.3 | 27.6 ± 7.7 |
| *Smoking status, number (%)*  Current | 18 (11) | 14 (15) | 1 (8) | 11 (7) | 9 (9) | 3 (21) |
| Past | 92 (55) | 40 (44) | 7 (54) | 50 (32) | 26 (27) | 7 (50) |
| Never | 56 (34) | 38 (41) | 5 (39) | 97 (51) | 61 (64) | 4 (29) |
| SBP, mean ± SD, mmHg | 147 ± 18 | 147 ± 18 | 146 ± 18 | 151 ± 18 | 151 ± 19 | 158 ± 34 |
| DBP, mean ± SD, mmHg | 82 ± 10 | 82 ± 12 | 82 ± 11 | 82 ± 11 | 82 ± 10 | 82 ± 12 |
| *CAD entry (%)*  Chronic stable angina | 68 (41) | 35 (38) | 7 (54) | 80 (51) | 60 (63) | 9 (64) |
| Unstable angina > 1 mo ago | 42 (25) | 18 (20) | 4 (31) | 17 (11) | 9 (9) | 1 (7) |
| Abnormal angiogram | 137 (83) | 72 (78) | 7 (54) | 81 (51) | 54 (56) | 7 (50) |
| Abnormal stress test | 45 (27) | 23 (25) | 3 (23) | 24 (15) | 11 (11) | 2 (14) |
| Myocardial infarction, number (%) | 74 (45) | 41 (45) | 8 (62) | 62 (39) | 38 (40) | 4 (29) |
| Hypertension, number (%) | 166 (100) | 92 (100) | 13 (100) | 158 (100) | 96 (100) | 14 (100) |
| Heart failure, number (%) | 9 (5) | 2 (2) | 0 (0) | 11 (7) | 5 (5) | 3 (21) |
| Diabetes, number (%) | 1 (1) | 3 (3) | 0 (0) | 3 (2) | 2 (2) | 0 (0) |
| Hormone replacement therapy, number (%) | 0 | 0 | 0 | 41 (26) | 26 (28) | 2 (14) |

**Supplemental Table 8. INVEST baseline characteristics by -45965 G>C and sex**

|  | **-45965 G/G male (n=209)** | **-45965 G/C male (n=82)** | **-45965 C/C male (n=9)** | **-45965 G/G female (n=206)** | **-45965 G/C female (n=82)** | **-45965 C/C female (n=9)** |
| --- | --- | --- | --- | --- | --- | --- |
| Age, mean ± SD, years | 69 ± 9 | 70 ± 8 | 65 ± 8 | 73 ± 10 | 72 ± 11 | 67 ± 10 |
| BMI, mean ± SD, kg/m2 | 28.6 ± 4.2 | 28.3 ± 4.9 | 29.8 ± 2.9 | 28.1 ± 6.1 | 28.1 ± 5.2 | 29.6 ± 6.9 |
| *Smoking status, number (%)*  Current | 24 (11) | 14 (17) | 1 (11) | 14 (7) | 9 (11) | 1 (11) |
| Past | 106 (51) | 42 (51) | 5 (56) | 64 (31) | 25 (30) | 3 (56) |
| Never | 79 (38) | 26 (32) | 3 (33) | 128 (62) | 48 (59) | 5 (33) |
| SBP, mean ± SD, mmHg | 146 ± 18 | 147 ± 19 | 148 ± 20 | 150 ± 20 | 153 ± 19 | 149 ± 11 |
| DBP, mean ± SD, mmHg | 82 ± 11 | 82 ± 10 | 84 ± 9 | 82 ± 10 | 83 ± 11 | 80 ± 7 |
| *CAD entry (%)*  Chronic stable angina | 84 (40) | 29 (35) | 4 (44) | 117 (57) | 39 (48) | 4 (44) |
| Unstable angina > 1 mo ago | 45 (22) | 25 (30) | 2 (22) | 20 (10) | 8 (10) | 2 (22) |
| Abnormal angiogram | 173 (83) | 64 (78) | 7 (78) | 113 (55) | 42 (51) | 6 (67) |
| Abnormal stress test | 60 (29) | 17 (21) | 2 (22) | 29 (14) | 11 (13) | 1 (11) |
| Myocardial infarction, number (%) | 92 (44) | 36 (44) | 6 (67) | 79 (38) | 38 (46) | 4 (44) |
| Hypertension, number (%) |  |  |  |  |  |  |
| Heart failure, number (%) | 6 (3) | 5 (6) | 1 (11) | 17 (8) | 3 (4) | 0 (0) |
| Diabetes, number (%) | 4 (2) | 1 (1) | 0 (0) | 2 (1) | 5 (6) | 0 (0) |
| Hormone replacement therapy, number (%) | 0 | 0 | 0 | 59 (29) | 17 (21) | 3 (33) |

Supplemental Table 9. INVEST baseline characteristics by 80 G>A and sex

|  | **80 G/G male (n=69)** | **80 G/A male (n=161)** | **80 A/A male (n=71)** | **80 G/G female (n=78)** | **80 G/A female (n=148)** | **80 A/A female (n=76)** |
| --- | --- | --- | --- | --- | --- | --- |
| Age, mean ± SD, years | 69 ± 8 | 69 ± 9 | 69 ± 10 | 71 ± 10 | 73 ± 10 | 74 ± 9 |
| BMI, mean ± SD, kg/m2 | 29.2 ± 4.3 | 27.9 ± 4.0 | 29.3 ± 5.0 | 27.9 ± 6.2 | 28.2 ± 5.2 | 28.3 ± 6.8 |
| *Smoking status, number (%)*  Current | 9 (13) | 22 (14) | 7 (10) | 9 (12) | 11 (7) | 5 (7) |
| Past | 37 (54) | 82 (51) | 37 (52) | 27 (35) | 44 (30) | 24 (32) |
| Never | 23 (33) | 57 (35) | 27 (38) | 42 (54) | 93 (63) | 47 (62) |
| SBP, mean ± SD, mmHg | 147 ± 18 | 146 ± 18 | 148 ± 19 | 150 ± 18 | 153 ± 20 | 150 ± 22 |
| DBP, mean ± SD, mmHg | 82 ± 9 | 81 ± 11 | 83 ± 12 | 83 ± 11 | 83 ± 10 | 80 ± 10 |
| *CAD entry (%)*  Chronic stable angina | 26 (38) | 58 (36) | 34 (48) | 49 (63) | 75 (51) | 41 (54) |
| Unstable angina > 1 mo ago | 21 (30) | 31 (19) | 18 (25) | 6 (8) | 14 (9) | 9 (12) |
| Abnormal angiogram | 57 (83) | 129 (80) | 57 (80) | 31 (40) | 87 (59) | 43 (57) |
| Abnormal stress test | 13 (19) | 42 (26) | 24 (34) | 11 (14) | 22 (15) | 9 (12) |
| Myocardial infarction, number (%) | 31 (45) | 74 (46) | 31 (44) | 33 (42) | 62 (42) | 28 (37) |
| Hypertension, number (%) | 69 (100) | 161 (100) | 71 (100) | 78 (100) | 148 (100) | 76 (100) |
| Heart failure, number (%) | 4 (6) | 4 (2) | 5 (7) | 4 (5) | 9 (6) | 7 (9) |
| Diabetes, number (%) | 1 (1) | 3 (2) | 1 (1) | 0 (0) | 5 (3) | 2 (3) |
| Hormone replacement therapy, number (%) | 0 (0) | 0 (0) | 0 (0) | 25 (32) | 36 (25) | 20 (26) |

Supplemental Table 10. INVEST baseline characteristics by M201T and sex

|  | **M201M male (n=282)** | **M201T male (n=23)** | **M201M female (n=271)** | **M201T female (n=27)** | **T201T female**  **(n=1)** |
| --- | --- | --- | --- | --- | --- |
| Age, mean ± SD, years | 69 ± 9 | 69 ± 10 | 72 ± 10 | 74 ± 10 | 84 |
| BMI, mean ± SD, kg/m2 | 28.6 ± 4.4 | 27.8 ± 4.1 | 28.1 ± 5.7 | 28.8 ± 7.3 | 18.1 |
| *Smoking status, number (%)*  Current | 36 (13) | 3 (13) | 22 (8) | 2 (7) | 0 (0) |
| Past | 147 (52) | 13 (57) | 85 (31) | 8 (30) | 1 (100) |
| Never | 99 (35) | 7 (30) | 164 (61) | 17 (63) | 0 (0) |
| SBP, mean ± SD, mmHg | 146 ± 18 | 148 ± 20 | 151 ± 20 | 152 ± 19 | 151 |
| DBP, mean ± SD, mmHg | 82 ± 11 | 80 ± 10 | 82 ± 10 | 82 ± 11 | 73 |
| *CAD entry (%)*  Chronic stable angina | 111 (39) | 8 (35) | 148 (55) | 15 (56) | 1 (100) |
| Unstable angina > 1 mo ago | 67 (24) | 5 (22) | 28 (10) | 2 (7) | 0 (0) |
| Abnormal angiogram | 224 (79) | 22 (96) | 146 (54) | 13 (48) | 0 (0) |
| Abnormal stress test | 74 (26) | 7 (30) | 39 (14) | 4 (15) | 0 (0) |
| Myocardial infarction, number (%) | 126 (45) | 12 (52) | 109 (40) | 11 (41) | 0 (0) |
| Hypertension, number (%) | 282 (100) | 23 (100) | 271 (100) | 27 (100) | 1 (100) |
| Heart failure, number (%) | 10 (4) | 2 (9) | 18 (7) | 2 (7) | 0 (0) |
| Diabetes, number (%) | 4 (1) | 1 (4) | 7 (3) | 0 (0) | 0 (0) |
| Hormone replacement therapy, number (%) | 0 (0) | 0 (0) | 71 (26) | 9 (33) | 0 (0) |

Supplemental Table 11. INVEST baseline characteristics by R264C and sex

|  | **R264R male (n=277)** | **R264C male (n=18)** | **C264C (n=1)** | **R264R female (n=271)** | **R264C female (n=26)** |
| --- | --- | --- | --- | --- | --- |
| Age, mean ± SD, years | 69 ± 9 | 71 ± 8 | 77 | 73 ± 10 | 73 ± 10 |
| BMI, mean ± SD, kg/m2 | 28.4 ± 4.4 | 29.1 ± 3.4 | 28.6 | 28.2 ± 5.9 | 27.9 ± 5.6 |
| *Smoking status, number (%)*  Current | 37 (13) | 1 (6) | 0 (0) | 23 (8) | 1 (4) |
| Past | 143 (52) | 11 (61) | 1 (100) | 83 (31) | 9 (35) |
| Never | 97 (35) | 1 (6) | 0 (0) | 165 (61) | 16 (62) |
| SBP, mean ± SD, mmHg | 146 ± 18 | 144 ± 16 | 142 | 151 ± 20 | 152 ± 17 |
| DBP, mean ± SD, mmHg | 82 ± 11 | 82 ± 11 | 83 | 82 ± 11 | 81 ± 11 |
| *CAD entry (%)*  Chronic stable angina | 111 (40) | 6 (33) | 0 (0) | 150 (55) | 13 (50) |
| Unstable angina > 1 mo ago | 60 (22) | 9 (50) | 0 (0) | 26 (10) | 4 (15) |
| Abnormal angiogram | 225 (81) | 15 (83) | 1 (100) | 144 (53) | 14 (54) |
| Abnormal stress test | 77 (28) | 5 (28) | 0 (0) | 39 (14) | 3 (12) |
| Myocardial infarction, number (%) | 124 (45) | 10 (56) | 0 (0) | 103 (38) | 16 (62) |
| Hypertension, number (%) | 277 (100) | 18 (100) | 1 (100) | 271 (100) | 26 (100) |
| Heart failure, number (%) | 9 (3) | 3 (17) | 0 (0) | 16 (6) | 2 (8) |
| Diabetes, number (%) | 5 (2) | 0 (0) | 0 (0) | 6 (2) | 1 (4) |
| Hormone replacement therapy, number (%) | 0 (0) | 0 (0) | 0 (0) | 70 (26) | 8 (32) |

Supplemental Table 12. INVEST baseline characteristics by R264C and sex

|  | **32266 G/G male (n=161)** | **32266 G/T male (n=120)** | **32266 T/T male (n=27)** | **32266 G/G female (n=159)** | **32266 G/T female (n=127)** | **32266 T/T female (n=17)** |
| --- | --- | --- | --- | --- | --- | --- |
| Age, mean ± SD, years | 69 ± 10 | 69 ± 9 | 68 ± 7 | 73 ± 9 | 72 ± 10 | 72 ± 8 |
| BMI, mean ± SD, kg/m2 | 29.0 ± 4.5 | 27.8 ± 4.2 | 29.0 ± 3.8 | 28.1 ± 6.2 | 28.4 ± 5.4 | 26.6 ± 5.5 |
| *Smoking status, number (%)*  Current | 17 (11) | 17 (14) | 6 (22) | 12 (8) | 11 (9) | 2 (12) |
| Past | 87 (54) | 61 (51) | 11 (41) | 46 (29) | 48 (38) | 2 (12) |
| Never | 57 (35) | 42 (35) | 10 (37) | 101 (64) | 68 (54) | 13 (77) |
| SBP, mean ± SD, mmHg | 146 ± 18 | 146 ± 17 | 153 ± 20 | 152 ± 20 | 151 ± 20 | 149 ± 16 |
| DBP, mean ± SD, mmHg | 82 ± 11 | 82 ± 11 | 82 ± 10 | 81 ± 10 | 83 ± 11 | 82 ± 7 |
| *CAD entry (%)*  Chronic stable angina | 61 (38) | 51 (43) | 9 (33) | 89 (56) | 62 (49) | 13 (76) |
| Unstable angina > 1 mo ago | 38 (24) | 28 (23) | 7 (26) | 20 (13) | 9 (7) | 1 (6) |
| Abnormal angiogram | 133 (83) | 93 (78) | 22 (81) | 86 (54) | 67 (53) | 9 (53) |
| Abnormal stress test | 45 (28) | 32 (27) | 4 (15) | 21 (13) | 20 (16) | 2 (12) |
| Myocardial infarction, number (%) | 71 (44) | 53 (44) | 14 (52) | 62 (39) | 58 (46) | 3 (18) |
| Hypertension, number (%) | 161 (100) | 120 (100) | 27 (100) | 159 (100) | 127 (100) | 17 (100) |
| Heart failure, number (%) | 9 (6) | 4 (3) | 0 (0) | 15 (9) | 4 (3) | 1 (6) |
| Diabetes, number (%) | 1 (1) | 3 (3) | 1 (4) | 4 (3) | 3 (2) | 0 (0) |
| Hormone replacement therapy, number (%) | 0 (0) | 0 (0) | 0 (0) | 39 (25) | 36 (29) | 6 (35) |

Supplemental Table 13. Case-control status by sex and genotype in INVEST.

| ***Males*** | | | |
| --- | --- | --- | --- |
|  | **-81371 C/C** | **-81371 C/T** | **-81371 T/T** |
| Case | 37 (23%) | 21 (23%) | 7 (54%) |
| Control | 125 (77%) | 69 (77%) | 6 (46%) |
| ***Females*** | | | |
| Case | 46 (29%) | 17 (18%) | 2 (14%) |
| Control | 112 (71%) | 77 (82%) | 12 (86%) |
| ***Males*** | | | |
|  | **-45965 G/G** | **-45965 G/C** | **-45965 C/C** |
| Case | 52 (25%) | 19 (23%) | 0 (0%) |
| Control | 152 (75%) | 62 (77%) | 9 (100%) |
| ***Females*** | | | |
| Case | 44 (21%) | 25 (31%) | 4 (44%) |
| Control | 161 (79%) | 56 (69%) | 5 (56%) |
| ***Males*** | | | |
|  | **80 G/G** | **80 G/A** | **80 A/A** |
| Case | 17 (25%) | 36 (23%) | 21 (30%) |
| Control | 52 (75%) | 119 (77%) | 50 (70%) |
| ***Females*** | | | |
| Case | 20 (26%) | 39 (27%) | 15 (20%) |
| Control | 58 (74%) | 107 (73%) | 61 (80%) |
| ***Males*** | | | |
|  | **M201M** | **M201T** | **T201T** |
| Case | 69 (25%) | 6 (27%) | 0 (0%) |
| Control | 208 (75%) | 16 (73%) | 0 (0%) |
| ***Females*** | | | |
| Case | 69 (26%) | 7 (26%) | 0 (0%) |
| Control | 200 (74%) | 20 (74%) | 1 (100%) |
|  |  |  |  |
| ***Males*** | | | |
|  | **R264R** | **R264C** | **C264C** |
| Case | 63 (23%) | 6 (33%) | 0 (0%) |
| Control | 209 (77%) | 12 (67%) | 1 (100%) |
| ***Females*** | | | |
| Case | 62 (23%) | 9 (36%) | 0 (0%) |
| Control | 209 (77%) | 16 (64%) | 0 (0%) |
| ***Males*** | | | |
|  | **32226 G/G** | **32226 G/T** | **32226 T/T** |
| Case | 38 (24%) | 31 (27%) | 7 (26%) |
| Control | 121 (76%) | 85 (73%) | 20 (74%) |
| ***Females*** | | | |
| Case | 42 (27%) | 27 (21%) | 5 (29%) |
| Control | 116 (73%) | 99 (79%) | 12 (71%) |

**Supplemental Figure 1.** Cumulative mortality incidence by -81371 C>T genotype and sex. Log rank p=0.60 in females and log rank p=0.24 in males.


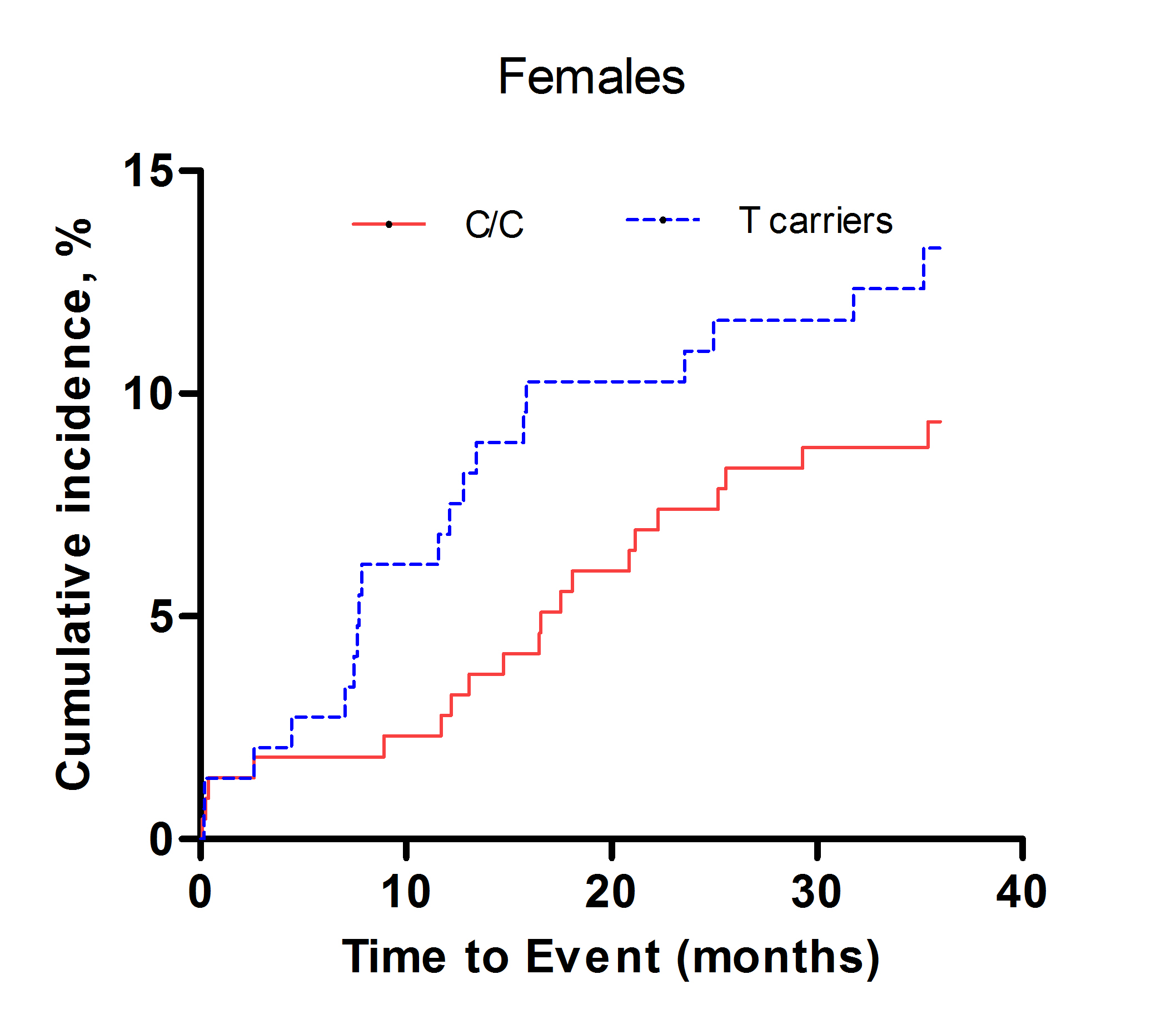

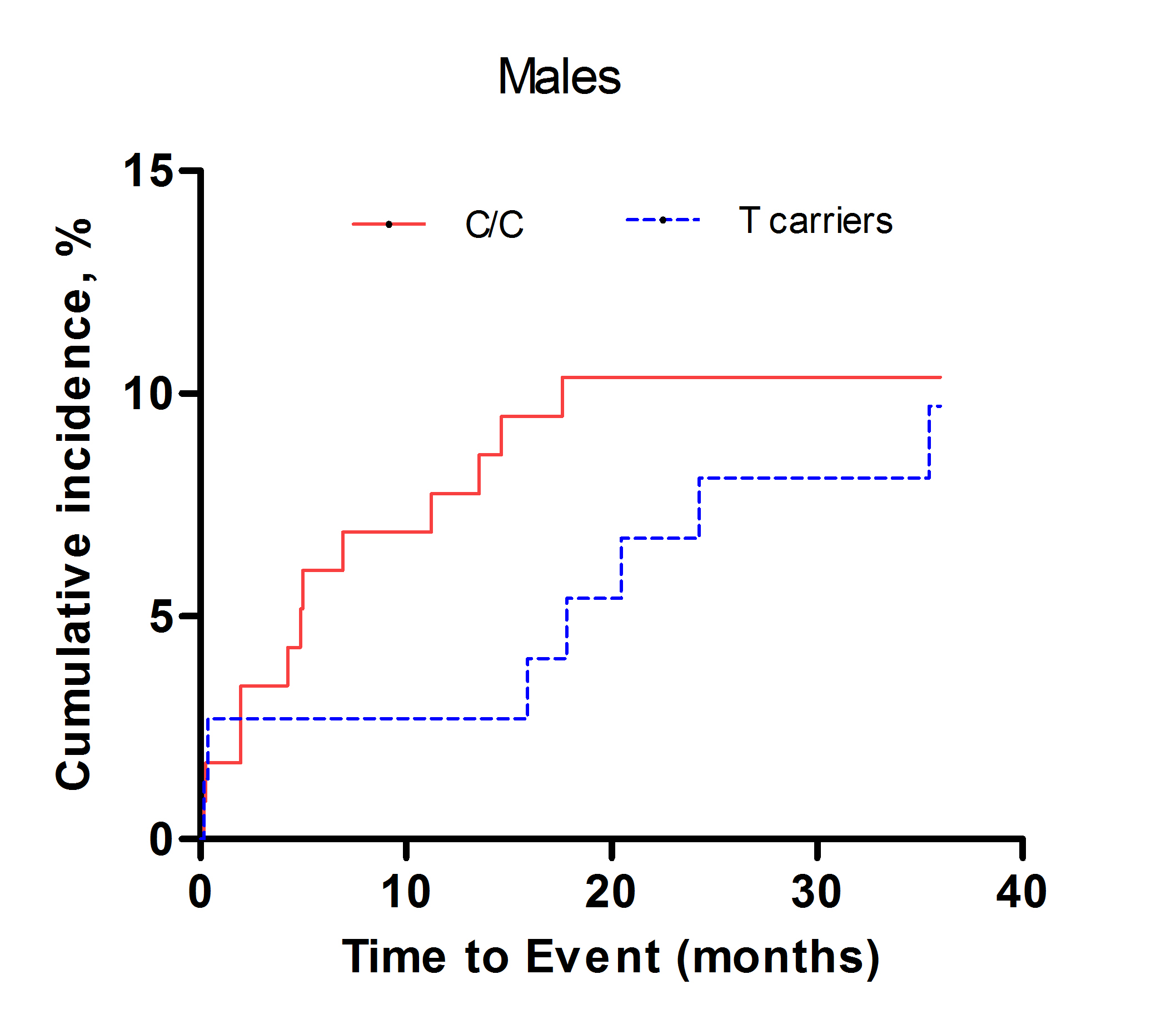

Supplement: File S1 — Includes tables of baseline characteristics by genotype and sex for INFORM and INVEST, table of case/control status by sex and genotype in INVEST, and Kaplan Meier plots of cumulative mortality incidence by -81371 C>T genotype and sex. (DOC) [file pone.0015180.s001.doc]
